# Depressive symptoms, oral health behaviors, and self-reported oral health conditions among Chinese university students: a cross-sectional study

**DOI:** 10.3389/froh.2026.1950461

**Published:** 2026-09-23

**Authors:** Wei Wang, Yanzhi Wang, Jingfen Zhang, Rui Li, Zifu Yu, Luxiang Shang, Xinyu Chen, Yuyan Yin

**Affiliations:** 1 Department of General Dentistry, Jinan Stomatological Hospital, Jinan, China; 2 Department of General Dentistry II, Jinan Stomatological Hospital, Jinan, China; 3 Shandong Center for Disease Control and Prevention, Jinan, China; 4 Department of Health Management, Shandong Engineering Research Center of Health Management, Shandong Institute of Health Management, the First Affiliated Hospital of Shandong First Medical University & Shandong Provincial Qianfoshan Hospital, Jinan, China

**Keywords:** cross-sectional study, depressive symptoms, oral health behaviors, self-rated oral health, self-reported oral health problems, university students

## Abstract

**Introduction:**

Depression has been associated with adverse oral health behaviors and conditions, but the mechanisms underlying these associations remain incompletely understood, particularly the potential role of oral health behaviors. This study examined these associations of depressive symptoms with oral health behaviors and self-reported oral health and explored statistical indirect associations through oral health behaviors among Chinese university students.

**Methods:**

This cross-sectional study included 2,695 undergraduate students from seven universities in Shandong, China. Data were collected using an online questionnaire. Depressive symptoms were assessed using the 10-item Center for Epidemiologic Studies Depression Scale (CESD-10). Oral health was self-reported and included self-rated oral health and six predefined oral health problems, including dental caries, gingival bleeding, oral ulcers, tooth sensitivity, oral malodor, and other periodontal-related problems. Ten favorable oral health behaviors were summarized using an unweighted composite score. Multivariable linear, multinomial logistic, negative binomial, and binary logistic regression models were used as appropriate. Exploratory mediation analyses with 5,000 bootstrap resamples assessed statistical indirect associations through oral health behaviors.

**Results:**

Higher CESD-10 scores were associated with poorer oral health behaviors and worse self-reported oral health. Each 1-point increase in CESD-10 score was associated with a lower oral health behavior score (*β* = −0.049, 95% CI: −0.063 to −0.036; *P* < 0.001) and a greater number of self-reported oral health problems (IRR = 1.026, 95% CI: 1.020–1.033; *P* < 0.001). For self-rated oral health, the strength of association increased progressively across worsening categories, with adjusted ORs ranging from 1.039 (95% CI: 1.010–1.069; *P* = 0.009) for good vs. very good oral health to 1.213 (95% CI: 1.154–1.274; *P* < 0.001) for very poor vs. very good oral health. Mediation analysis showed significant statistical indirect associations through oral health behaviors, accounting for 14.41% of the association with self-rated oral health (95% CI: 9.73%–20.30%; *P* < 0.001) and 5.76% of the association with the number of oral health problems (95% CI: 2.17%–10.79%; *P* = 0.0004).

**Conclusion:**

Depressive symptoms were associated with poorer oral health behaviors, worse self-rated oral health, and a greater burden of self-reported oral health problems among university students. Oral health behaviors explained a modest proportion of these statistical associations. These findings support further investigation of integrated mental and oral health promotion strategies in university populations.

## Introduction

Depression is a common mental health disorder and has become a major public health concern worldwide, particularly among young adults (1). Beyond its psychological consequences, depression has been increasingly recognized as an important determinant of physical health through its effects on health behaviors, self-care practices, and healthcare utilization (2). In the field of oral health, previous studies have reported that individuals with depression or depressive symptoms are more likely to engage in suboptimal oral health behaviors, including inadequate tooth brushing, less frequent use of dental floss, and reduced utilization of dental care services (3). Furthermore, depressive symptoms have been associated with poorer oral health status and a greater burden of oral diseases (4, 5). Therefore, understanding the relationship between depressive symptoms and oral health may provide important insights for developing integrated strategies addressing both mental and oral health.

Although depression is clinically defined as a psychiatric disorder, depressive symptoms represent a broader spectrum of psychological distress that can be measured dimensionally in population-based studies. Previous epidemiological studies have demonstrated that depressive symptoms, even in individuals without a formal diagnosis of depression, are associated with impaired self-care behaviors and adverse health outcomes (2, 6). Accordingly, assessing depressive symptoms using validated screening instruments may help identify psychological factors associated with oral health in general populations, particularly in non-clinical settings.

Despite increasing evidence linking depressive symptoms with oral health, several knowledge gaps remain. First, previous studies have often focused on individual oral health behaviors or specific oral conditions, whereas oral health behaviors represent a multidimensional construct involving daily hygiene practices, use of oral care products, and preventive dental service utilization. A comprehensive evaluation of multiple oral health behaviors may provide a broader understanding of how depressive symptoms are associated with oral health practices (7, 8). Second, although depressive symptoms and oral health are known to be associated, the extent to which oral health behaviors contribute to this association remains unclear. In particular, empirical evidence evaluating whether oral health behaviors statistically account for part of the relationship between depressive symptoms and oral health outcomes is limited, especially among non-clinical young adults and Chinese populations.

University students represent a particularly relevant population for investigating these associations. This life stage involves a transition from family-dependent health management to greater individual responsibility for health-related decisions, while also being accompanied by academic stress, changes in sleep patterns, dietary habits, and lifestyle behaviors (9). At the same time, students increasingly make independent decisions regarding preventive oral care and dental service utilization. Therefore, psychological status during this period may be closely related to daily oral health practices and perceived oral health.

Therefore, this study aimed to examine the associations between depressive symptoms and oral health conditions among Chinese university students. Specifically, we investigated the relationships of depressive symptoms with individual oral health behaviors, an overall oral health behavior composite score, self-rated oral health status, and self-reported oral health problems. Furthermore, we explored whether oral health behaviors statistically accounted for part of the associations between depressive symptoms and oral health outcomes.

## Material and methods

### Study design and population

This cross-sectional study was conducted in 2024 among undergraduate students from universities in Shandong Province, eastern China. The overall study framework, sampling strategy, and data collection procedures have been described in detail in our previous publication (10). In brief, participants were recruited through multiple channels, including campus-based announcements and social media platforms. A total of 2,805 questionnaires were distributed. After excluding incomplete responses and invalid questionnaires, 2,695 eligible undergraduate students from seven universities completed the self-administered online questionnaire via the Wenjuanxing platform and were included in the final analysis.

The study was approved by the Ethics Committee of Jinan Stomatological Hospital (No. JNSKQYY-2024-026). Electronic informed consent was obtained from all participants prior to participation. No personally identifiable information was collected, and all data were handled anonymously and confidentially.

### Assessment of depressive symptoms

Depressive symptoms were assessed using the Chinese version of the 10-item Center for Epidemiologic Studies Depression Scale (CESD-10) (Supplementary Table S1). The CESD-10 is a widely used screening instrument for evaluating depressive symptom severity in epidemiological studies. The Chinese version has demonstrated acceptable reliability and validity in previous studies, with reported Cronbach's *α* values ranging from 0.78 to 0.79) (11), and has been widely applied in large-scale population-based investigations, including the China Health and Retirement Longitudinal Study (CHARLS) (12, 13). The Cronbach's *α* coefficient for the CESD-10 scale was 0.845 in our study, indicating good internal consistency.

Each item is rated on a four-point Likert scale ranging from 0 to 3, generating a total score from 0 to 30. Two positively worded items were reverse-coded prior to analysis. Higher CESD-10 scores indicate more severe depressive symptoms. In this study, CESD-10 was used as a continuous measure of depressive symptom severity rather than a diagnostic tool for major depressive disorder.

### Assessment of oral health behaviors and self-reported oral health measures

Demographic characteristics, oral health-related behaviors, self-rated oral health status, and self-reported oral health problems were collected using the structured questionnaire.

Oral health behaviors were assessed using 10 indicators: brushing teeth ≥2 times/day, brushing for ≥2 min per session, use of dental floss, use of fluoride toothpaste, use of mouthwash, toothbrush replacement within 3 months, brushing after late-night snacking, proper brushing technique, regular professional dental scaling, and routine dental check-ups. The selection of these indicators was based on clinical experience, feedback obtained from university students during questionnaire development, and oral health behaviors commonly evaluated in previous studies among young adults (14–16). These indicators were designed to cover multiple aspects of oral health practices, including daily oral hygiene, use of auxiliary oral care products, and preventive dental service utilization. Each behavior was coded as 1 when the predefined favorable criterion was met and 0 otherwise, and the 10 items were summed without differential weighting to generate a composite score ranging from 0 to 10. Equal weighting was used because no established weighting scheme exists for these heterogeneous oral health behaviors in university students. A higher score indicates engagement in a greater number of favorable oral health practices. This score was developed as a pragmatic formative summary index for the present study and has not been independently validated as a psychometric scale.

Self-rated oral health status was assessed using a single-item question: “How would you rate your oral health?”. Participants selected one of five response categories ranging from “very good” to “very poor,” which were scored from 1 to 5, with higher scores indicating poorer perceived oral health.

In addition, participants were asked whether they had experienced any of six common oral health problems: dental caries, gingival bleeding, oral ulcers, tooth sensitivity, oral malodor, and periodontal-related problems (e.g., tooth mobility). These conditions were selected because they represent common oral health problems frequently reported among adolescents and young adults and have been widely investigated in epidemiological studies (17). All oral health problems were self-reported based on participants' previous experiences or current symptoms. Each reported problem was coded as 1, and absent problems were coded as 0. The total number of self-reported oral health problems was calculated by summing the six individual problems, resulting in a count outcome ranging from 0 to 6.

### Data analysis

All statistical analyses were conducted using SPSS version 25.0 and R version 4.5.2. Continuous variables are presented as means ± standard deviations (SD), while categorical variables are expressed as proportions. Participants were stratified into tertiles according to CESD-10 scores. Differences in demographic characteristics, oral health behaviors, and self-reported oral health measures across CESD-10 tertiles were evaluated using one-way analysis of variance (ANOVA) and chi-square tests. The internal consistency of the CESD-10 scale was assessed using Cronbach's *α* coefficient.

Multivariable regression analyses were performed to evaluate the associations between depressive symptoms and oral health measures. The selection of regression models was based on the characteristics and distribution of each outcome variable. Multivariable binary logistic regression models were used to examine associations between CESD-10 scores and individual oral health behaviors as well as individual self-reported oral health problems. The composite oral health behavior score was analyzed using multivariable linear regression with HC3 heteroskedasticity-robust standard errors. Self-rated oral health status was analyzed using multivariable multinomial logistic regression, with “very good” oral health as the reference category. The number of self-reported oral health problems was analyzed using negative binomial regression, and robust Poisson regression was conducted as a sensitivity analysis. All multivariable models were adjusted for age, sex, grade, paternal education level, maternal education level, and place of residence. Results were presented as *β* coefficients, odds ratios (ORs), or incidence rate ratios (IRRs) with corresponding 95% confidence intervals (CIs), according to the applied regression models.

Exploratory mediation analyses were conducted using the “mediation” package in *R* to evaluate whether oral health behaviors statistically accounted for part of the associations between depressive symptoms and oral health measures. In these analyses, CESD-10 score was specified as the exposure, the composite oral health behavior score as the mediator, and self-rated oral health status or the number of self-reported oral health problems as outcomes. Indirect, direct, and total associations were estimated using 5,000 nonparametric bootstrap resamples with 95% CIs. A two-sided *P* value <0.05 was considered statistically significant.

## Results

### Participant characteristics by CESD-10 tertiles

Participants were categorized into tertiles according to CESD-10 scores (Table 1). 802 participants were classified as T1 (scores 0–3), 958 as T2 (scores 4–8), and 935 as T3 (scores ≥9). Mean CESD-10 scores were 1.22, 5.95, and 12.93 across T1, T2, and T3, respectively. Compared with T1, participants in T3 differed in grade distribution and were more likely to have lower paternal educational levels, whereas age, sex, place of residence, and maternal education did not differ significantly across tertiles.

**Table 1:** Comparison of sociodemographic characteristics by CESD-10 tertiles.

Characteristic	T1 (*n* = 802)	T2 (*n* = 958)	T3 (*n* = 935)	*P* value
Age	19.07 ± 1.10	18.97 ± 1.16	18.95 ± 1.18	0.068
Male	254 (31.67)	304 (31.73)	305 (32.62)	0.889
Grade				0.004
1	339 (42.27)	440 (45.93)	461 (49.30)	
2	312 (38.90)	355 (37.06)	343 (36.68)	
3	124 (15.46)	121 (12.63)	89 (9.52)	
4 or 5	27 (3.37)	42 (4.38)	42 (4.49)	
Place of residence				0.090
Urban	428 (53.37)	479 (50.00)	450 (48.13)	
Rural	374 (46.63)	479 (50.00)	485 (51.87)	
Father's education level				0.004
Primary school or below	64 (7.98)	115 (12.00)	110 (11.76)	
Middle school	286 (35.66)	357 (37.27)	384 (41.07)	
High school	248 (30.92)	275 (28.71)	246 (26.31)	
University and above	204 (25.44)	211 (22.03)	195 (20.86)	
Mother's education level				0.267
Primary school or below	135 (16.83)	183 (19.10)	189 (20.21)	
Middle school	290 (36.16)	351 (36.64)	359 (38.40)	
High school	215 (26.81)	253 (26.41)	235 (25.13)	
University and above	162 (20.20)	171 (17.85)	152 (16.26)	
CESD-10 score	1.22 ± 1.20	5.95 ± 1.24	12.93 ± 3.74	<0.001
Oral health behavior score	4.92 ± 2.01	4.63 ± 1.95	4.13 ± 1.95	<0.001
Self-rated oral health status				<0.001
1 (very good)	128 (15.96)	128 (13.36)	53 (5.67)	
2 (good)	290 (36.16)	260 (27.14)	228 (24.39)	
3 (fair)	327 (40.77)	457 (47.70)	477 (51.02)	
4 (poor)	51 (6.36)	97 (10.13)	141 (15.08)	
5 (very poor)	6 (0.75)	16 (1.67)	36 (3.85)	
Number of self-reported oral health problems	1.16 ± 1.22	1.31 ± 1.23	1.64 ± 1.46	<0.001

### Association between depressive symptoms and oral health behaviors

Across specific oral health behaviors, several favorable practices became less common with increasing CESD-10 tertile, including brushing teeth ≥2 times/day, brushing for ≥2 min per session, timely toothbrush replacement, brushing after late-night snacking, proper brushing technique, and preventive dental care practices (all *P* < 0.05; see Supplementary Table S2). Multivariable binary logistic regression analyses yielded largely consistent results (Figure 1A), showing that higher levels of depressive symptoms were associated with a lower likelihood of engaging in most oral health behaviors. Associations with floss use, mouthwash use, and proper brushing technique were not statistically significant.

**Figure 1:**
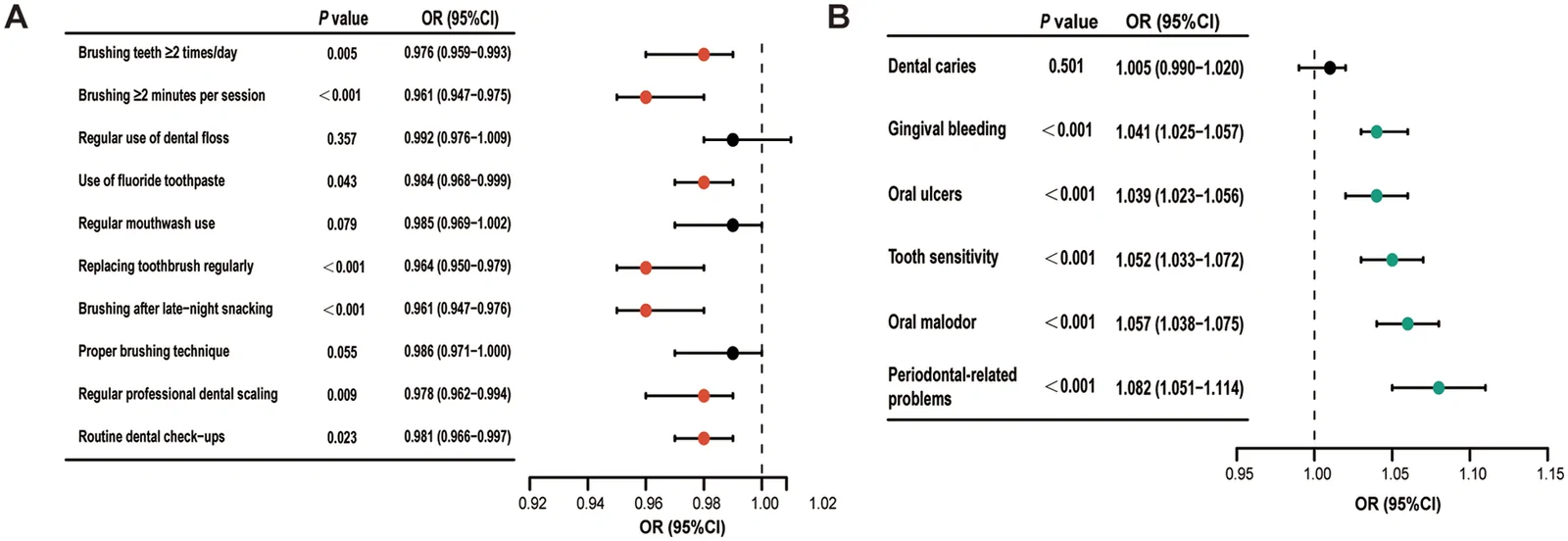
Forest plots showing the associations between CESD-10 score and oral health outcomes. **(A)** Associations between CESD-10 score and oral health behaviors. **(B)** Associations between CESD-10 score and oral health problems. Results are presented as odds ratios with 95% confidence intervals from logistic regression models.

For the composite measure, higher CESD-10 score was independently associated with a lower oral health behavior score in the multivariable linear regression model with HC3 robust standard errors (*β* = −0.049, 95% CI: −0.063 to −0.036; *P* < 0.001; Table 2). Female students had higher behavior scores than male students (*β* = 0.546, 95% CI: 0.388–0.705; *P* < 0.001), and higher scores were also observed among students in Grade 3 and Grades 4–5 and among those with higher maternal educational attainment, as shown in Table 2.

**Table 2:** Multivariable regression analyses of factors associated with oral health behavior score and number of self-reported oral health problems.

Variables	Outcome = Oral health behavior score		Outcome = Number of oral health problems	
	*β* (95%CI)	*P*	IRR (95%CI)	*P*
CESD-10 score, per 1-point increase	−0.049 (−0.063 to −0.036)	<0.001	1.026 (1.020–1.033)	<0.001
Age, per 1-year increase	−0.043 (–0.145 to 0.059)	0.406	0.971 (0.922–1.023)	0.273
Female	0.546 (0.388–0.705)	<0.001	1.326 (1.223–1.438)	<0.001
Grade				
1	Reference		Reference	
2	0.083 (−0.106 to 0.273)	0.389	1.027 (0.935–1.128)	0.576
3	0.457 (0.161–0.754)	0.003	1.020 (0.876–1.188)	0.801
4 or 5	1.351 (0.780–1.921)	<0.001	0.866 (0.652–1.150)	0.319
Mother's education level				
Primary school or below	Reference		Reference	
Middle school	0.368 (0.156–0.579)	<0.001	0.904 (0.815–1.002)	0.055
High school	0.559 (0.299–0.819)	<0.001	0.850 (0.748–0.966)	0.013
University and above	0.996 (0.678–1.315)	<0.001	0.834 (0.713–0.976)	0.023
Father's education level				
Primary school or below	Reference		Reference	
Middle school	−0.019 (−0.278 to 0.239)	0.883	1.036 (0.914–1.175)	0.580
High school	0.073 (−0.216 to 0.362)	0.622	1.029 (0.892–1.186)	0.698
University and above	−0.068 (−0.397 to 0.261)	0.688	0.938 (0.795–1.106)	0.445
Reside in rural areas	−0.139 (−0.308 to 0.030)	0.107	1.048 (0.963–1.140)	0.278

### Association between depressive symptoms and self-rated oral health and oral health problems

Self-rated oral health worsened progressively across CESD-10 tertiles, accompanied by an increasing number of self-reported oral health problems (both *P* < 0.001; Table 1). At the specific oral problem level, higher depressive symptom levels were associated with higher odds of gingival bleeding, oral ulcers, tooth sensitivity, oral malodor, and periodontal-related problems (all *P* < 0.001, Figure 1B and Supplementary Table S3). In contrast, CESD-10 score was not significantly associated with self-reported dental caries (OR = 1.005, 95% CI: 0.990–1.020; *P* = 0.501).

In multinomial logistic regression, CESD-10 score was significantly associated with self-rated oral health overall (likelihood-ratio *χ*^2^ = 117.26, *P* < 0.001; Table 3). The magnitude of association increased progressively across worsening categories: for each 1-point increase in CESD-10 score, the adjusted OR was 1.039 (95% CI: 1.010–1.069; *P* = 0.009) for good vs. very good oral health, 1.083 (95% CI: 1.054–1.113; *P* < 0.001) for fair vs. very good, 1.143 (95% CI: 1.106–1.182; *P* < 0.001) for poor vs. very good, and 1.213 (95% CI: 1.154–1.274; *P* < 0.001) for very poor vs. very good. In the negative binomial model, each 1-point increase in CESD-10 score was associated with a 2.6% higher expected number of self-reported oral health problems (IRR = 1.026, 95% CI: 1.020–1.033; *P* < 0.001; Table 2). A robust Poisson sensitivity analysis yielded an almost identical estimate (ratio = 1.026, 95% CI: 1.019–1.033; *P* < 0.001).

**Table 3:** Multivariable multinomial logistic regression analysis of factors associated with self-rated oral health.

Variable	Global *P*	Good vs. Very good		Fair vs. Very good		Poor vs. Very good		Very poor vs. Very good	
		OR (95% CI)	*P*	OR (95% CI)	*P*	OR (95% CI)	*P*	OR (95% CI)	*P*
CESD-10 score, per 1-point increase	<0.001	1.039 (1.010–1.069)	0.009	1.083 (1.054–1.113)	<0.001	1.143 (1.106–1.182)	<0.001	1.213 (1.154–1.274)	<0.001
Age, per 1-year increase	0.027	0.868 (0.720–1.048)	0.140	1.010 (0.846–1.206)	0.915	0.778 (0.612–0.990)	0.041	0.820 (0.536–1.253)	0.359
Female	<0.001	1.839 (1.402–2.412)	<0.001	2.551 (1.966–3.310)	<0.001	3.597 (2.492–5.194)	<0.001	2.055 (1.116–3.786)	0.021
Grade	0.089								
1		Reference		Reference		Reference		Reference	
2		0.964 (0.678–1.371)	0.837	0.940 (0.672–1.315)	0.719	1.099 (0.711–1.699)	0.671	0.553 (0.256–1.194)	0.132
3		1.196 (0.692–2.066)	0.522	0.848 (0.502–1.433)	0.538	1.138 (0.559–2.316)	0.722	0.393 (0.089–1.746)	0.220
4 or 5		0.798 (0.327–1.946)	0.620	0.334 (0.142–0.789)	0.012	0.612 (0.172–2.185)	0.450	NE	NE
Mother's education level	0.007								
Primary school or below		Reference		Reference		Reference		Reference	
Middle school		0.504 (0.315–0.807)	0.004	0.567 (0.361–0.889)	0.013	0.431 (0.254–0.730)	0.002	0.320 (0.143–0.716)	0.006
High school		0.642 (0.371–1.110)	0.113	0.602 (0.355–1.021)	0.060	0.369 (0.194–0.702)	0.002	0.278 (0.098–0.793)	0.017
University and above		0.493 (0.266–0.913)	0.025	0.414 (0.229–0.750)	0.004	0.212 (0.099–0.454)	<0.001	0.163 (0.044–0.603)	0.007
Father's education level	0.238								
Primary school or below		Reference		Reference		Reference		Reference	
Middle school		0.889 (0.509–1.553)	0.680	0.954 (0.561–1.623)	0.863	0.891 (0.476–1.668)	0.718	0.349 (0.148–0.820)	0.016
High school		1.170 (0.636–2.155)	0.614	1.022 (0.570–1.833)	0.942	0.888 (0.438–1.798)	0.741	0.319 (0.112–0.908)	0.032
University and above		0.848 (0.439–1.641)	0.625	0.803 (0.427–1.508)	0.495	0.867 (0.396–1.900)	0.721	0.262 (0.075–0.916)	0.036
Reside in rural areas	0.823	1.035 (0.751–1.428)	0.832	1.013 (0.746–1.376)	0.932	1.125 (0.758–1.671)	0.559	0.753 (0.385–1.472)	0.407

NE, not estimable.

### Exploratory analysis of statistical indirect associations

Exploratory mediation analyses with 5,000 bootstrap resamples indicated statistically significant indirect associations through the oral health behavior composite score (see Supplementary Table S4). For self-rated oral health, the indirect association was 0.00489 (95% CI: 0.00329–0.00657; *P* < 0.001), accounting for 14.41% (95% CI: 9.73%–20.30%; *P* < 0.001) of the overall association. For the number of self-reported oral health problems, the indirect association was 0.00217 (95% CI: 0.00082–0.00372; *P* = 0.0004), corresponding to 5.76% (95% CI: 2.17%–10.79%; *P* < 0.001) of the overall association. A sensitivity analysis using a Poisson outcome model yielded a similar proportion of 6.13% (95% CI: 2.31%–11.44%; *P* = 0.0004). These estimates represent statistical indirect associations and do not establish temporal or causal mediation.

## Discussion

In this cross-sectional study of 2,695 Chinese university students, higher depressive symptom levels were associated with poorer overall oral health behaviors, progressively worse categories of self-rated oral health, and a greater number of self-reported oral health problems. Associations were also observed for several specific behaviors and oral health problems, whereas no clear association was found with self-reported dental caries. Exploratory mediation analysis further suggested modest statistical indirect associations through oral health behaviors, which were more pronounced for self-rated oral health than for the number of oral health problems.

The inverse association between depressive symptoms and oral health behaviors is consistent with previous evidence (18). Depressive symptoms may be accompanied by reduced motivation, fatigue, and difficulties with attention or executive functioning, which can interfere with regular self-care (19, 20). In the present study, higher CESD-10 scores were associated with lower odds of several daily and preventive practices, including frequent and sufficiently long toothbrushing, timely toothbrush replacement, brushing after late-night snacking, professional dental scaling, and routine dental check-ups. However, the associations were not uniform across all practices; floss use, mouthwash use, and proper brushing technique were not significantly associated with CESD-10 score after multivariable adjustment. These findings suggest that the relationship between depressive symptoms and oral health practices may vary across different types of behaviors rather than affecting all behaviors to the same extent.

Beyond behavioral patterns, higher depressive symptom levels were associated with worse self-rated oral health and a greater burden of self-reported oral health problems. Previous studies have suggested that the relationship between oral health and depressive symptoms may be bidirectional (21, 22). Several mechanisms proposed in previous literature, including stress-related biological responses, immune alterations, and sleep disturbance, could plausibly contribute to these associations (19, 23–25); however, none of these mechanisms was directly measured in the present study. The absence of a significant association with self-reported dental caries should also be interpreted cautiously. Although the chronic and cumulative development of caries may make it less responsive to contemporaneous psychological variation, misclassification from self-report and the absence of a clinical oral examination may also have attenuated the observed association. These possibilities should therefore be regarded as explanatory hypotheses rather than mechanisms demonstrated by our data.

The exploratory mediation analysis suggested that oral health behaviors statistically accounted for only a modest proportion of the associations between depressive symptoms and oral health measures. The estimated proportion was larger for self-rated oral health (14.41%) than for the number of self-reported oral health problems (5.76%). One possible explanation is that subjective health appraisal may be more closely related to current daily behaviors, whereas the accumulation of oral health problems reflects a broader set of biological, behavioral, and healthcare-related factors over time (26–28). Nevertheless, this interpretation remains tentative. Because depressive symptoms, behaviors, and oral health measures were assessed at the same time, the analysis cannot establish that depressive symptoms preceded behavioral changes or that behavioral changes preceded oral health outcomes; reverse and bidirectional relationships remain possible.

These findings may have implications for future health promotion among university students, a population in which psychological stress and independent health-management behaviors often coexist. Rather than supporting immediate implementation of a specific screening or intervention program, our results suggest that mental health considerations may be relevant when designing oral health promotion strategies for university populations. Prospective studies are needed to determine whether depressive symptoms predict subsequent changes in oral health behaviors and oral health, and interventional studies should evaluate whether integrated mental and oral health approaches provide measurable benefits.

### Strengths and limitations

A major strength of this study is the relatively large multi-university sample and the simultaneous evaluation of multiple oral health behaviors and self-reported oral health measures using outcome-appropriate statistical models. Several limitations should also be considered. First, the cross-sectional design precludes determination of temporal sequence or causality and allows the possibility of reverse or bidirectional associations. This limitation is particularly important for the exploratory mediation analysis, which identifies statistical indirect associations but does not establish a causal pathway. Second, all oral health measures were self-reported, and no clinical oral examination was performed; therefore, misclassification and reporting bias are possible, especially for conditions such as dental caries and periodontal-related problems. Third, CESD-10 measures depressive symptom severity rather than providing a clinical diagnosis of depressive disorder, and no diagnostic interview or biological markers were available (29). Fourth, the oral health behavior score was an unweighted formative composite of heterogeneous behaviors rather than a validated unidimensional psychometric scale; its modest internal consistency (Cronbach's *α* = 0.519) should therefore be considered when interpreting the composite result. Fifth, the sample was limited to university students from one Chinese province, which may restrict generalizability to other geographic regions or non-student populations. Finally, although major demographic confounders were adjusted for, residual confounding from unmeasured socioeconomic, psychological, dietary, or healthcare-related factors cannot be excluded.

## Conclusion

Depressive symptoms were associated with poorer oral health behaviors, progressively worse self-rated oral health, and a greater number of self-reported oral health problems among Chinese university students. Exploratory analyses suggested modest statistical indirect associations through oral health behaviors, particularly for self-rated oral health. These findings support further longitudinal and interventional research to clarify temporal relationships and evaluate integrated mental and oral health promotion strategies in university populations.

## Data Availability

The raw data supporting the conclusions of this article will be made available by the authors, without undue reservation.
