# Supplementary material for: Depressive symptoms, oral health behaviors, and self-reported oral health conditions among Chinese university students: a cross-sectional study
Source: Front Oral Health. 2026 Sep 23;7:1950461. doi: 10.3389/froh.2026.1950461 (PMC13642022; doi:10.3389/froh.2026.1950461)
Supplement: Supplementary file 1 [file Datasheet1.docx]

**Depressive symptoms, oral health behaviors, and self-reported oral health conditions among Chinese university students: A cross-sectional study**

**Supplementary files:**

Supplementary Table S1. CESD-10 questions and answers.

Supplementary Table S2. Distribution of oral health behaviors by CESD-10 tertiles.

Supplementary Table S3. Comparison of oral health problems by CESD-10 tertiles.

Supplementary Table S4. Exploratory mediation analysis of depressive symptoms, oral health behaviors, and oral health measures.

**Supplementary Table S1****. CESD-10 questions and answers**

The 10 items below refer to how you have felt and behaved during the last week. Choose the appropriate response.

Questions

1．I was bothered by things that don’t usually bother me.

2．I had trouble keeping my mind on what I was doing.

3．I felt depressed.

4．I felt everything I did was an effort.

5．I felt hopeful about the future. （positive one）

6．I felt fearful.

7．My sleep was restless.

8．I was happy. （positive one）

9．I felt lonely.

10．I could not get “going”.

Answers

1．Rarely or none of the time (< 1 day)
2．Some or a little of the time (1-2 days)
3．Occasionally or a moderate amount of the time (3-4 days)
4．Most or all of the time (5-7 days)

**Supplementary Table S2. Distribution of oral health behaviors by CES-D-10 tertiles**

| Characteristic | T1 (n = 802) | T2 (n = 958) | T3 (n = 935) | *P* value |
| --- | --- | --- | --- | --- |
| Daily brushing frequency |  |  |  | <0.001 |
| 0-1 times | 153 (19.08) | 214 (22.34) | 252 (26.95) |  |
| 2 times or more | 649 (80.92) | 744 (77.66) | 683 (73.05) |  |
| Brushing duration per session |  |  |  | <0.001 |
| Less than 2 minutes | 269 (33.54) | 341 (35.59) | 435 (46.52) |  |
| More than 2 minutes | 533 (66.46) | 617 (64.41) | 500 (53.48) |  |
| Regular use of dental floss |  |  |  | 0.158 |
| No | 569 (70.95) | 711 (74.22) | 699 (74.76) |  |
| Yes | 233 (29.05) | 247 (25.78) | 236 (25.24) |  |
| Use of fluoride toothpaste |  |  |  | 0.098 |
| No or not sure | 551 (68.70) | 672 (70.15) | 685 (73.26) |  |
| Yes | 251 (31.30) | 286 (29.85) | 250 (26.74) |  |
| Regular use of mouthwash |  |  |  | 0.075 |
| No | 579 (72.19) | 696 (72.65) | 715 (76.47) |  |
| Yes | 223 (27.81) | 262 (27.35) | 220 (23.53) |  |
| Frequency of toothbrush replacement |  |  |  | <0.001 |
| Longer than 3 months | 226 (28.18) | 319 (33.30) | 371 (39.68) |  |
| Once within 3 months | 576 (71.82) | 639 (66.70) | 564 (60.32) |  |
| Brushing after a late-night snack |  |  |  | <0.001 |
| No | 218 (27.18) | 298 (31.11) | 373 (39.89) |  |
| Yes | 584 (72.82) | 660 (68.89) | 562 (60.11) |  |
| Brushing technique |  |  |  | 0.002 |
| Horizontal brushing | 430 (53.62) | 527 (55.01) | 574 (61.39) |  |
| Vertical brushing | 372 (46.38) | 431 (44.99) | 361 (38.61) |  |
| Professional scaling frequency |  |  |  | 0.003 |
| Never or less than once a year | 544 (67.83) | 682 (71.19) | 704 (75.29) |  |
| At least once a year | 258 (32.17) | 276 (28.81) | 231 (24.71) |  |
| Routine dental check-ups |  |  |  | 0.013 |
| Never or less than once a year | 535 (66.71) | 683 (71.29) | 683 (73.05) |  |
| At least once a year | 267 (33.29) | 275 (28.71) | 252 (26.95) |  |

**Supplementary Table S3. Comparison of oral health problems by CES-D-10 tertiles**

| Characteristic | T1 (n = 802) | T2 (n = 958) | T3 (n = 935) | *P* value |
| --- | --- | --- | --- | --- |
| Dental caries, n (%) |  |  |  | 0.070 |
| No | 498 (62.09) | 545 (56.89) | 542 (57.97) |  |
| Yes | 304 (37.91) | 413 (43.11) | 393 (42.03) |  |
| Gingival bleeding, n (%) |  |  |  | <0.001 |
| No | 609 (75.94) | 699 (72.96) | 604 (64.60) |  |
| Yes | 193 (24.06) | 259 (27.04) | 331 (35.40) |  |
| Oral ulcers, n (%) |  |  |  | <0.001 |
| No | 623 (77.68) | 715 (74.63) | 631 (67.49) |  |
| Yes | 179 (22.32) | 243 (25.37) | 304 (32.51) |  |
| Tooth sensitivity, n (%) |  |  |  | <0.001 |
| No | 682 (85.04) | 834 (87.06) | 729 (77.97) |  |
| Yes | 120 (14.96) | 124 (12.94) | 206 (22.03) |  |
| Oral malodor, n (%) |  |  |  | <0.001 |
| No | 687 (85.66) | 792 (82.67) | 703 (75.19) |  |
| Yes | 115 (14.34) | 166 (17.33) | 232 (24.81) |  |
| Periodontal diseases, n (%) |  |  |  | <0.001 |
| No | 782 (97.51) | 909 (94.89) | 865 (92.51) |  |
| Yes | 20 (2.49) | 49 (5.11) | 70 (7.49) |  |

**Supplementary Table S4.** **Exploratory mediation analysis of depressive symptoms, oral health behaviors, and oral health measures**

| Outcome | Indirect association | *P* | Direct association | *P* | Total association | *P* | Proportion accounted for, % | *P* |
| --- | --- | --- | --- | --- | --- | --- | --- | --- |
| Self-rated oral health | 0.00489 (0.00329–0.00657) | <0.001 | 0.02903 (0.02230–0.03587) | <0.001 | 0.03392 (0.02716–0.04077) | <0.001 | 14.41 (9.73–20.30) | <0.001 |
| Number of self-reported oral health problems (Linear model) | 0.00217 (0.00082–0.00372) | 0.0004 | 0.03545 (0.02506–0.04631) | <0.001 | 0.03762 (0.02744–0.04839) | <0.001 | 5.76 (2.17–10.79) | 0.0004 |
| Number of self-reported oral health problems (Poisson model) | 0.00184 (0.00069–0.00320) | 0.0004 | 0.02822 (0.02118–0.03460) | <0.001 | 0.03006 (0.02334–0.03622) | <0.001 | 6.13 (2.31–11.44) | 0.0004 |
